# Eco-physiological adaptation shapes the response of calcifying algae to nutrient limitation

**DOI:** 10.1038/srep16499

**Published:** 2015-11-12

**Authors:** Luka Šupraha, Andrea C. Gerecht, Ian Probert, Jorijntje Henderiks

**Affiliations:** 1 Paleobiology, Department of Earth Sciences, Uppsala University, Villavägen 16, 75236 Uppsala, Sweden; 2 CEES, Department of Biosciences, University of Oslo, P.O. Box 1066 Blindern, 0316 Oslo, Norway; 3 UPMC, CNRS, Roscoff Biological Station, Place Georges Teissier, 29680 Roscoff, France

## Abstract

The steady increase in global ocean temperature will most likely lead to nutrient limitation in the photic zone. This will impact the physiology of marine algae, including the globally important calcifying coccolithophores. Understanding their adaptive patterns is essential for modelling carbon production in a low-nutrient ocean. We investigated the physiology of *Helicosphaera carteri*, a representative of the abundant but under-investigated flagellated functional group of coccolithophores. Two strains isolated from contrasting nutrient regimes (South Atlantic and Mediterranean Sea) were grown in phosphorus-replete and phosphorus-limited batch cultures. While growing exponentially in a phosphorus-replete medium, the Mediterranean strain exhibited on average 24% lower growth rate, 36% larger coccosphere volume and 21% lower particulate inorganic carbon (PIC) production than the Atlantic strain. Under phosphorus limitation, the same strain was capable of reaching a 2.6 times higher cell density than the Atlantic strain due to lower phosphorus requirements. These results suggest that local physiological adaptation can define the performance of this species under nutrient limitation.

Coccolithophores, the dominant calcifying component of marine phytoplankton, exhibit complex interactions with the biogeochemical carbon cycle^1^. Their net contribution to carbon flux is determined as a balance among rates of calcification (CO_2_ source), photosynthesis (CO_2_ sink) and export production from surface waters^1^,^2^,^3^. The scale of these three processes depends on physiological traits of coccolithophores, such as cell size and cellular production rates, which can be modified by environmental forcing^4^. Climate warming models predict that rising ocean surface temperature will enhance stratification of the water column, thereby decreasing nutrient supply to the photic zone^5^,^6^,^7^. On a large scale, changes in nutrient availability will affect the biogeographic distribution^8^, community structure^9^ and primary production^10^ of modern phytoplankton. To model the performance of coccolithophores under lower nutrient availability, it is essential to identify their nutrient-related adaptive traits.

A number of experimental investigations have addressed the response of coccolithophores to limitation by nutrients, particularly phosphorus and nitrogen. Only non-motile coccolithophores have been investigated to date, these being ecologically important but representing only a small portion of the more than 280 coccolithophore species^11^. Most of the previous work has focused on the model species *Emiliania huxleyi*^12^,^13^,^14^,^15^,^16^,^17^,^18^, with recent studies on *Calcidiscus leptoporus*^19^ and *Coccolithus pelagicus*^20^,^21^. The available data indicates that nutrient limitation can have a strong effect on cell size and carbon production^22^. Previous investigations have also shown that the response of the group to nutrient limitation is strongly constrained by species-specific and even strain-specific physiological traits. However, the origin of strain-specific responses is not yet fully understood, and no clear link to local eco-physiological adaptation has been experimentally confirmed^15^.

The present study used batch culture experiments to detect phosphorus-related eco-physiological traits in *Helicosphaera carteri*. This species is a flagellated, motile coccolithophore, representing a functionally prevalent but under-investigated form within the group (Supplementary Fig. 1). In its diploid life cycle stage, *H. carteri* produces large, heavily calcified heterococcoliths, and thus has a significant potential for affecting carbon fluxes^2^,^23^. Having large cells and two orders-of-magnitude higher phosphorus requirements than *E. huxleyi*^15^, this species is likely to experience strong selective pressure under reduced phosphorus availability. Nevertheless, the geological record of the *H. carteri* morphospecies extends over the past 23 Ma^24^, indicating that the morphospecies has a considerable adaptive potential to global environmental changes. In this context, *H. carteri* strains that inhabit oligotrophic areas of the modern oceans are presumably adapted to low phosphorus levels. As such, they can provide insights into the physiological traits that may occur under future low-nutrient conditions.

The two strains of *H. carteri* used in this study were isolated from contrasting nutrient regimes (Fig. 1, Supplementary Table 1). The Atlantic strain was isolated from the nutrient-rich Southern Benguela upwelling area, where phosphorus is less likely to limit phytoplankton growth than nitrogen or silica^25^. The Mediterranean strain was isolated from the Western Mediterranean (Villefranche-sur-Mer Bay), an oligotrophic area where surface phosphate concentrations are commonly below the detection level^26^. The basic physiology of the strains was investigated under phosphorus-replete conditions to investigate the intrinsic eco-physiological traits linked to phosphorus availability at the isolation site. The strains were then exposed to phosphorus (P-) limitation to test whether strain-specific eco-physiological traits affect the response pattern. Finally, partial 28 S rDNA sequences of the strains were compared to determine whether strain-specific traits represent cryptic speciation or intra-specific ecophenotypic plasticity and genetic variability.

## Results

### Strain-specific traits in *H. carteri*

**Figure 2:**
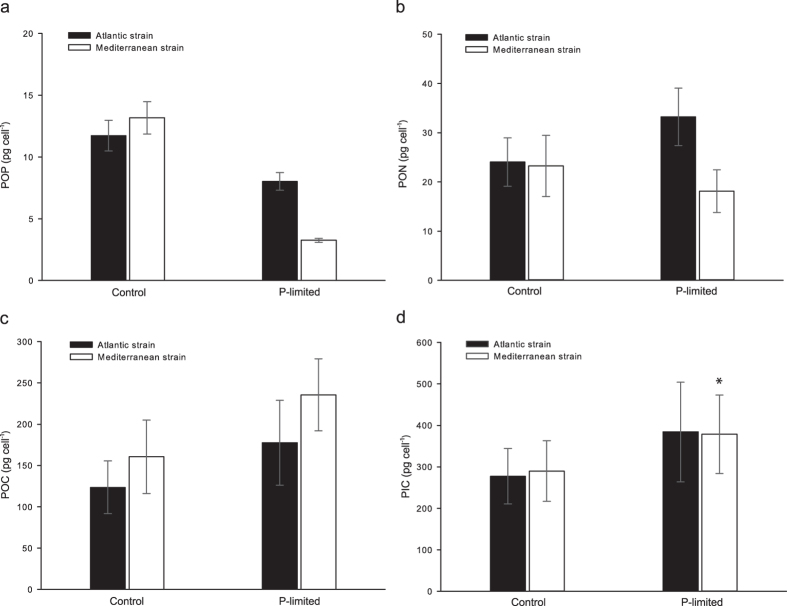
Cellular quotas of POP, PON, POC and PIC measured at the end of the control (high-P) and P-limited experiments. Each bar represents pooled measurements from three replicates. Two samples were taken from each replicate (total N = 6). The asterisk (*) highlights the batch culture with 2.6-fold higher cell density and altered carbonate chemistry. Error bars show standard deviation.

**Figure 3:**
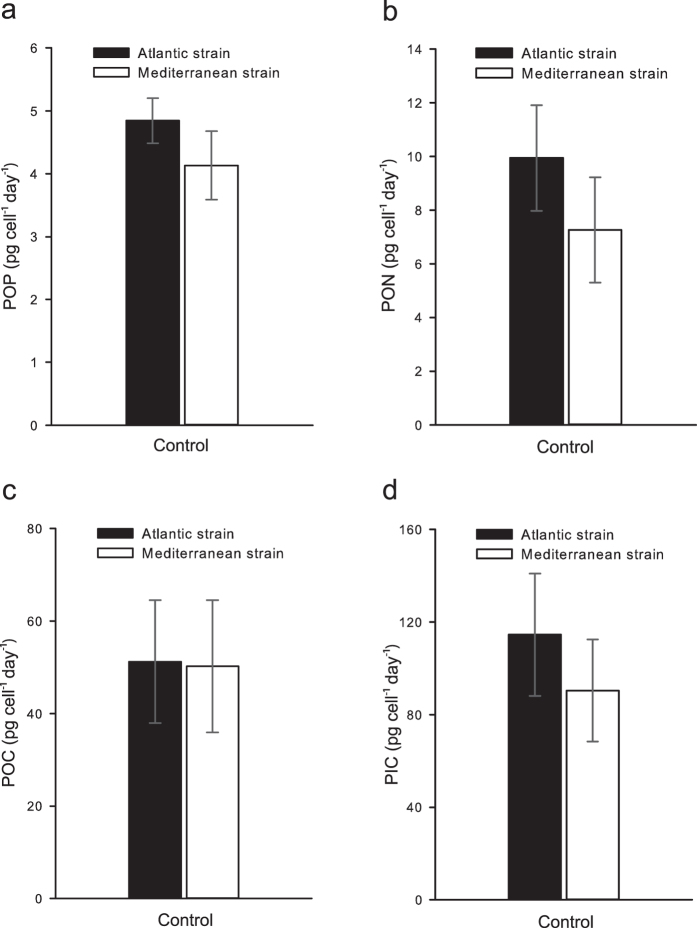
Cellular production rates of POP, PON, POC and PIC in the control cultures. Each bar represents pooled measurements from the three replicates. Two samples were taken from each replicate (total N = 6). Error bars show standard deviation.

Our experiments demonstrated that physiological and morphological differences do exist between strains of *H. carteri* (unpaired t-tests). During exponential growth in phosphorus-replete (high-P) medium, the strains exhibited clearly distinct cell and coccosphere size (Supplementary Fig. 2, Supplementary Fig. 3). The Mediterranean strain was larger both in terms of mean cell (+36%; t = −10.99, df = 4, p < 0.001) and mean coccosphere volume (+36%; t = −7.37, df = 4, p < 0.001). The mean coccosphere volumes of the two strains were inversely related to their mean growth rates (μ), and the larger Mediterranean strain grew on average 24% slower (μ = 0.31 day^−1^) than the Atlantic strain (μ = 0.41 day^−1^; t = −6.76, df = 4, p = 0.001; Supplementary Fig. 3, Supplementary Table 2). The two strains showed significant differences in the rate of nutrient acquisition. Although the cellular quota of particulate organic phosphorus (POP) was 12% higher in the Mediterranean than in the Atlantic strain (t = −1.95, df = 10, p = 0.040; Fig. 2a), POP production was 15% lower (t = 2.44, df = 10, p = 0.017; Fig. 3a). The production of particulate organic nitrogen (PON) was 27% lower in the Mediterranean strain (t = 1.72, df = 10, p = 0.058; Fig. 3b) while the PON quota was similar (t = 0.22, df = 10, p = 0.414; Fig. 2b). Particulate organic carbon (POC) quota scaled with cell size, being on average 30% higher in the Mediterranean strain (t = −1.66 df = 10, p = 0.064; Fig. 2c). Despite the lower growth rate in the Mediterranean strain, POC production (rate of photosynthesis) was similar between the strains (t = 0.13, df = 10, p = 0.450; Fig. 3c). There was no significant difference in particulate inorganic carbon (PIC) quota (t = −0.30, df = 10, p = 0.385; Fig. 2d), even though cells of the Mediterranean strain were on average covered with 1–2 more coccoliths than cells of the Atlantic strain (Supplementary Fig. 4). Individual coccolith measurements revealed no significant difference in mean coccolith volume between the strains (t = 1.46, df = 329, p = 0.072; Supplementary Fig. 5). Still, the PIC production (calcification rate) was 21% lower in the Mediterranean strain (t = 2.15, df = 10, p = 0.028; Fig. 3d). This lower PIC production resulted in a 20% lower PIC/POC production ratio (t = 2.50, df = 10, p = 0.016), moving the biogeochemical balance of this strain towards carbon uptake. However, the PIC/POC ratio of the Mediterranean strain was sufficiently high (>1.6) for the strain to continue acting as a net source of CO_2_^27^.

### Phosphorus physiology and response pattern

**Figure 4:**
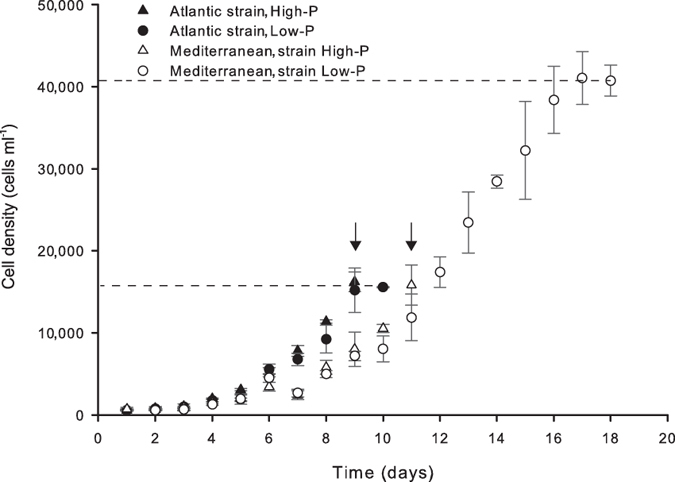
Cell density of the two strains during the course of the experiment. Each data point represents the average of three replicates. Each replicate value represents an average of all measurements performed by the particle counter. Error bars represent the standard deviation of the three replicates. Arrows indicate the density at which exponentially growing cells from the control cultures (high-P) were sampled. Dotted lines mark the maximum cell abundance reached by the strains in the low-P treatments before the onset of the stationary phase.

The difference in maximum cell density reached by the two strains in low-phosphorus (low-P) medium confirmed our hypothesis that strain-specific physiological traits can affect their response to P-limitation (paired t-tests, unless indicated otherwise). Although the strains consumed the same amount of phosphorus (unpaired t-test, t = −0.34, df = 10, p = 0.740) before entering the stationary phase, they had reached significantly different final cell densities (Supplementary Table 3). The Atlantic strain entered the stationary phase at a density of 15,590 cells ml^−1^, while the Mediterranean strain grew exponentially for 8 additional days reaching 40,780 cells ml^−1^, *i.e.* a 2.6 times higher cell density (Fig. 4). The performance of the Mediterranean strain under P-limitation can be explained by the POP quota which is an indicator of minimum cellular phosphorus requirements. The Mediterranean strain had a 2.5-fold lower POP quota (i.e. phosphorus requirements) than its Atlantic counterpart (Fig. 2a), which allowed it to reach a higher cell density under P-limitation. The PON quota of the Atlantic strain increased by 38% under P-limitation (t = −2.80, df = 5, p = 0.019; Fig. 2b), mirroring cell volume and POC increases (Fig. 2c). In contrast, in the P-limited Mediterranean strain, PON quota decreased by an average of 22% (t = 3.32, df = 5, p = 0.011; Fig. 2b), despite the increase in cell volume and POC quota.

A common physiological response of coccolithophores to P-limitation at the onset of stationary phase includes a reduction in growth rate, reduction of POP quota, an increase in cell size and accumulation of POC^15^,^19^,^20^. All of these responses were observed in our P-limited batch cultures (Fig. 2a, c, Supplementary Fig. 2). The incremental growth rate of the Atlantic strain dropped from 0.5 day^−1^ on day 9 to 0.03 day^−1^ on day 10 when the cells were sampled. The growth rate of the Mediterranean strain was declining gradually towards the end of the experiment, reaching a negative rate of −0.01 day^−1^ on the sampling day (Supplementary Fig. 2). Reduction in POP quota was more pronounced in the Mediterranean strain (75% lower under P-limitation; t = 17.92, df = 5, p < 0.001) compared to the Atlantic strain (32% lower than control; t = 5.19, df = 5, p = 0.002; Fig. 2a). Coccosphere size increased sharply in the Atlantic strain, with a 19% increase on day 10 (Supplementary Fig. 2). The increase in coccosphere volume was more continuous in the Mediterranean strain, which was gradually increasing in volume while approaching the stationary phase (Supplementary Fig. 2). The volume increase was accompanied by an increase in POC quota of both strains, which was 44% higher in the P-limited cells of the Atlantic strain compared to the control (t = −2.36, df = 5, p = 0.032), and 47% higher than the control in the P-limited Mediterranean strain (t = −21.37, df = 5, p < 0.001; Fig. 2c). Increased cell size allowed more coccoliths to be placed around the cell surface, which increased the PIC quota of both strains under P-limitation (Fig. 2d). Individual coccolith measurements showed that the mean coccolith volume did not change significantly in the Atlantic strain under P-limitation (Supplementary Fig. 4). However, a decrease in coccolith volume was observed in the P-limited Mediterranean strain. Coccolith volume, as well as the PIC quota in this culture, were likely underestimated due to the altered carbonate chemistry at the time of sampling, caused by the high cell density of the low-P Mediterranean batch cultures (Supplementary Fig. 5, Supplementary Table 3).

### Molecular analysis

The 739 bp long sequences generated from the studied strains were aligned with other 28 S rDNA sequences from Genbank (release 205, December 2014) assigned to haptophytes. Sequences from both strains used in this study were identical to a sequence assigned to *H. carteri* (accession number EU729473 from strain RCC1333) and differed by 2 bp from a sequence assigned to the closely related morphospecies *H. wallichii* (FJ696920 from RCC1328). The accession numbers of the sequences obtained in this study can be found in the Supplementary Table 1.

## Discussion

Our data suggests that P-limitation, prevalent in the modern Mediterranean, has imposed a selection pressure on the local strain of *H. carteri*, leading to modified phosphorus physiology. This strain-specific physiology was clearly visible when strains were compared under low-P conditions. Unlike the faster growing Atlantic strain, the Mediterranean strain reduced its phosphorus requirements to reach higher cell densities under the same level of P-limitation. From an evolutionary perspective, decreased phosphorus requirements may prove beneficial under nutrient limitation, providing strains from oligotrophic areas with an increased competitive ability. Adaptation to low phosphorus availability by decreasing phosphorus requirement is clearly an effective way of overcoming nutrient stress, as this morphospecies has been present worldwide throughout the major climate changes of the Cenozoic^28^. Interestingly, we observed a reduction in nitrogen quota in the P-limited Mediterranean strain, suggesting that the strain-specific physiology extends beyond phosphorus utilization, and that this strain can also reduce its nitrogen requirements under P-limitation. The same response was not observed in the Atlantic strain, where the nitrogen quota increased under P-limitation. This reduction in nitrogen utilization under P-limitation has never before been observed in coccolithophores, arguably because this study was the first one to investigate a large coccolithophore from an oligotrophic area in which such physiological traits are more likely to occur^19^,^20^.

The differences in phosphorus physiology were reflected in other physiological traits of the two strains, most notably cell size, growth rate and calcification rate. Growth rate and coccosphere size were negatively correlated among the strains, as is commonly the case in coccolithophores^29^,^30^. Reduced POP production of the exponentially growing Mediterranean strain was likely a result of reduced phosphorus requirements. Still, the strain kept the same POC production as the faster-growing Atlantic strain. This allocation of the limiting resource (phosphorus) to POC production led to a reduction in the rates of other key physiological processes, such as cell division rate and calcification. A similar response to P-limitation, with resource allocation to POC production and a reduction in growth rate and PIC production, has been observed in *C. pelagicus* in a semi-continuous experimental setup^21^. Slower division rates under the same rate of POC production resulted in larger cell size of the Mediterranean strain. Furthermore, the large cell size and the large volume to surface-area ratio (V/SA) of the Mediterranean strain likely contributed to reduced phosphorus uptake and POP production of the strain. In context of adaptation, this suggests that the adaptive strategy in this species is to reduce its phosphorus requirements under P-limitation rather than to reduce the cell size (and thereby the V/SA), which would arguably be favourable for nutrient uptake^31^,^32^.

Molecular analysis of the two strains provided strong evidence that the strains belong to the same species. Therefore, the strain-specific physiological traits represent an ecophenotypic plasticity or intraspecific genetic variability rather than cryptic speciation, the latter being common within the group^33^. Since the strains used in this study belong to the same species, their strain-specific adaptive traits could have developed either through ecophenotypic plasticity (gene regulation) or genetic variability (positive mutations). Adaptation experiments with *E. huxleyi* have shown that this coccolithophore can adapt to changing carbonate chemistry and increased temperature within one year of growing outside of its optimum conditions^34^. Regulation of gene expression was identified as an important part of the adaptation process in this case^35^. Other studies on *E. huxleyi* have argued that favourable mutations are an important adaptive mechanism^36^. The *H. carteri* strains used in our study had preserved their strain-specific adaptive traits after years of growing under optimum conditions in a culture collection. We therefore argue that long-term adaptation in *H. carteri* is strongly genetically constrained and is related to favourable mutations of genes controlling the phosphorus metabolism. This observation also confirms that traits do not change or evolve significantly without a selective pressure. The lack of a strain-specific response to P-limitation reported for *E. huxleyi* strains from the Mediterranean Sea^15^ can be attributed to the same principle. It is unlikely for a species already capable of blooming in a low phosphorus environment^4^ to develop strain-specific traits related to a nutrient that is not imposing a significant selective pressure.

Changes in carbon production under nutrient-limited exponential growth can only be tested in a continuous or semi-continuous experimental setup^19^. Nevertheless, from our exponentially growing batch cultures, we can infer the following about the effects of adaptation to P-limitation on the cellular balance of carbon release and uptake. The POC production of both strains was the same, despite different POP production. The conserved POC production in the Mediterranean strain was likely supported at the expense of lower cell division and calcification rates. Therefore, our findings suggests that in the long term, adaptive evolution in low-P environments could lead to a decrease in PIC production and the PIC/POC production ratio in *H. carteri*, as was observed in the Mediterranean strain. However, it is unlikely that the PIC/POC production ratio will decrease below the threshold for changing the carbon uptake-to-release balance of this species.

## Methods

### Helicosphaera carteri strains and medium preparation

Preliminary investigations were condu-cted on four strains of *H. carteri* which were obtained from the Roscoff Culture Collection (Supplementary Table 1). Three of the Atlantic strains showed significantly lower cell size than the Mediterranean strain, and the Atlantic strain RCC1323 was randomly selected for the comparison with the Mediterranean strain RCC1334 in the final experiment. Strains were grown in K/2 medium^37^, modified to favour the growth of calcifying algae (preparation details in ref. 20). High-P medium (10 μM) was used as a control treatment. A low-P medium (1 μM) induced P-limitation and led cells into stationary phase.

### Experimental setup

Before inoculation, strains were acclimated to the experimental conditions for approximately 10 generations. Stable conditions were ensured by growing cultures in an environmental test chamber (MLR-350, Panasonic, Japan), at a temperature of 17 °C and an irradiance of ~160 μmol m^−2^ s^−1^ under a 14:10 h light:dark cycle. During the experiment, strains were grown in triplicate in high-P and low-P medium. 350 ml volume cultures were inoculated at an initial concentration of 500 cells ml^−1^. Daily counts of cell abundance were performed using an electronic particle counter (CASY, Roche, Switzerland), two hours after the onset of the light phase. Exponential growth rates (μ) were calculated daily by linear regression of log-transformed cell density data. Low-P cultures were harvested once the cells had reached the stationary phase. High-P treatments were harvested during the exponential growth phase at similar cell density to ensure comparable carbonate chemistry.

### Sample analyses

Coccosphere size was measured daily as part of the automated cell density measurements. Cell size was measured using the same instrument, after the addition of 20 μl of 1 M HCl to 10 ml of sample, which dissolved the coccolith cover. Samples for the analysis of initial phosphate concentration in low-P medium were collected from the medium before the inoculation of cells, while samples for the analysis of residual phosphate, pH, and total alkalinity (A_T_) were collected during final harvesting of the cultures. For the residual phosphate analysis, the medium was sterile-filtered and stored at −20 °C until analysis. Phosphate concentration was measured using the colorimetric method^38^, ^39^, ^40^,^38^, ^39^, ^40^. For the analysis of pH and A_T_, sterile-filtered medium was stored at 4 °C in acid washed glass vials. Manual titration and pH measurements were performed within 24 h after sampling using a two-point calibrated combined electrode (Red Rod, Radiometer, Denmark). The carbonate chemistry was calculated from the pH and A_T_ inputs using the CO2sys program v2.1 (developed for MS Excel by D. Pierrot from CO2SYS.BAS Basic Program by E. Lewis and D. W. R. Wallace). For POP analysis, culture material was filtered onto pre-combusted GF/C filters and stored at −20 °C. POP was converted to orthophosphate (following the protocol in ref. 31) and analysed along with the residual phosphate samples. Samples for total particulate carbon (TC), POC and PON were filtered onto pre-combusted GF/C filters, dried in an oven at 60 °C and analysed on an elemental analyser (Flash 1112, Thermo Finnegan, USA). Filters for POC analysis were treated with 230 μL of 2 M HCl to dissolve the particulate inorganic carbon (PIC). PIC quota was determined subsequently as the difference between TC and POC. Cellular production rates were determined only in the exponentially growing high-P treatments as a product of growth rate and the corresponding cellular elemental quotas. Morphological analysis of coccoliths was performed with a Zeiss Supra35-VP scanning electron microscope. The analysis of individual coccolith volume followed the methodology described in ref. 40.

### Statistical analysis

One-tailed t-tests were performed using the Systat v13.1 software (Systat software Inc., USA). Paired t-tests were applied for within-strain comparisons and unpaired t-tests (labeled “two-sample t-test” in the Systat software) for between-strain comparisons. Differences with p-values < 0.05 were considered statistically significant.

### Molecular analysis

Genomic DNA was extracted from cultures harvested in the exponential growth phase using the DNeasy Plant mini kit (Qiagen, Netherlands) according to the manufacturer’s instructions. Partial 28 S ribosomal DNA (rDNA) genes were PCR amplified using the forward primer Leuk2F (5′-acccgctgaacttaagcatatcact-3′) and the reverse primer Leuk_34r (5′-gcatcgccagttctgcttacc-3′). PCRs were performed in a total reaction volume of 25 μL using the Phusion high-fidelity PCR master mix with GC buffer (Finnzymes, Finland). The PCR protocol employed was as follows: 30 s initial denaturation at 98 °C, followed by 30 cycles of 10 s at 98 °C, 30 s annealing at 55 °C and 1 min extension at 72 °C. A final 5-min extension step at 72 °C was conducted to complete the amplification. Amplification products were controlled by electrophoresis on a 1% agarose gel. PCR products were sequenced directly on an ABI PRISM 3100 xl DNA autosequencer (Perkin-Elmer Inc., USA) using the ABI PRISM BigDye Terminator Cycle Sequencing Kit (Perkin-Elmer). Alignments were generated using MUSCLE implemented in MEGA v6.06^41^ with subsequent manual verification.

## Additional Information

**How to cite this article**: Šupraha, L. *et al.* Eco-physiological adaptation shapes the response of calcifying algae to nutrient limitation. *Sci. Rep.* **5**, 16499; doi: 10.1038/srep16499 (2015).

## Supplementary Material

Supplementary Information

## Figures and Tables

**Figure 1:**
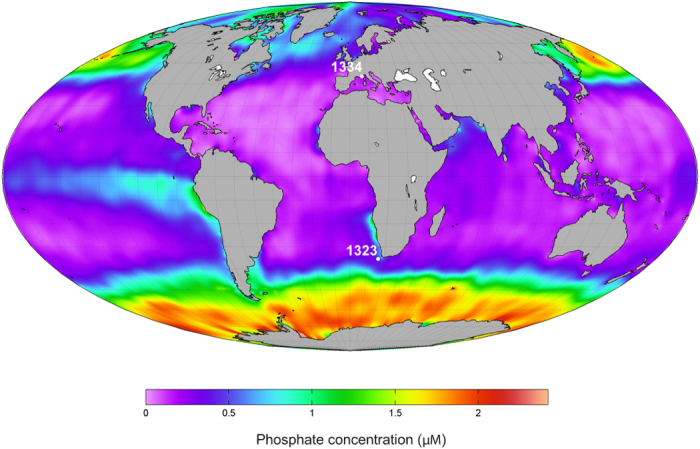
Annual mean surface phosphate concentrations and isolation sites of the strains used in this study. Data was obtained from the publicly available World Ocean Atlas 2013 database, provided by the National Oceanic and Atmospheric Administration (NOAA, www.nodc.noaa.gov/OC5/WOA09/netcdf_data.html)^42^. The distribution of phosphate was drawn in the Mollweide projection, using M_Map, a mapping package for Matlab freely available at http://www.eos.ubc.ca/~rich/map.html. The original 1° data were interpolated into a 0.25° grid.
