# Supplementary material for: Eco-physiological adaptation shapes the response of calcifying algae to nutrient limitation
Source: Sci Rep. 2015 Nov 12;5:16499. doi: 10.1038/srep16499 (PMC4642353; doi:10.1038/srep16499)
Supplement: Supplementary Information [file srep16499-s1.pdf]

Eco-physiological adaptation shapes the response of calcifying algae to nutrient limitation

SUPPLEMENTARY INFORMATION

Luka Šupraha[a*], Andrea C. Gerecht[b†], Ian Probert[c], Jorijntje Henderiks[a, b]

[a] Paleobiology, Department of Earth Sciences, Uppsala University, Villavägen 16, 75236 Uppsala, Sweden

[b] CEES, Department of Biosciences, University of Oslo, P.O. Box 1066 Blindern, 0316 Oslo, Norway

[c] UPMC, CNRS, Roscoff Biological Station, Place Georges Teissier, 29680 Roscoff, France

[†]Current address: UiT – The Arctic University of Norway, P.O. Box 6050 Langnes, 9037 Tromsø, Norway

*corresponding author: luka.supraha@geo.uu.se

This file contains:

Supplementary Figure 1

Supplementary Figure 2

Supplementary Figure 3

Supplementary Figure 4

Supplementary Figure 5

Supplementary Table 1

Supplementary Table 2

Supplementary Table 3

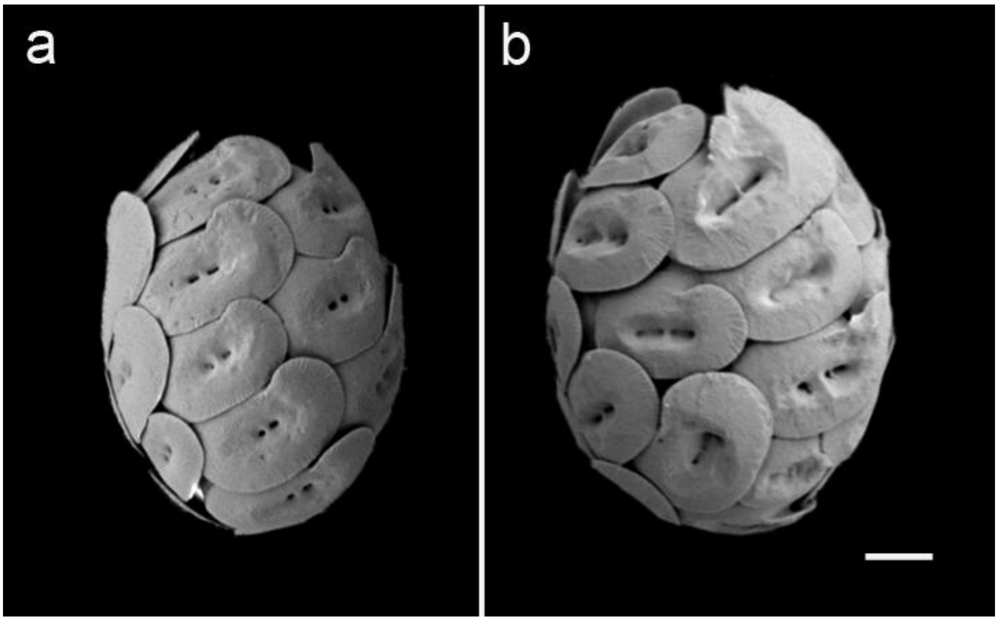

Supplementary Figure 1. SEM micrographs of *Helicosphaera carteri*. Note the characteristic helically arranged coccoliths (helicoliths) with the flagellar opening at the apical end of the coccosphere; a: Specimen obtained from the Atlantic strain (RCC 1323) control culture; B: Specimen obtained from the Mediterranean strain (RCC 1334) control culture. Scale bar: 3µm.

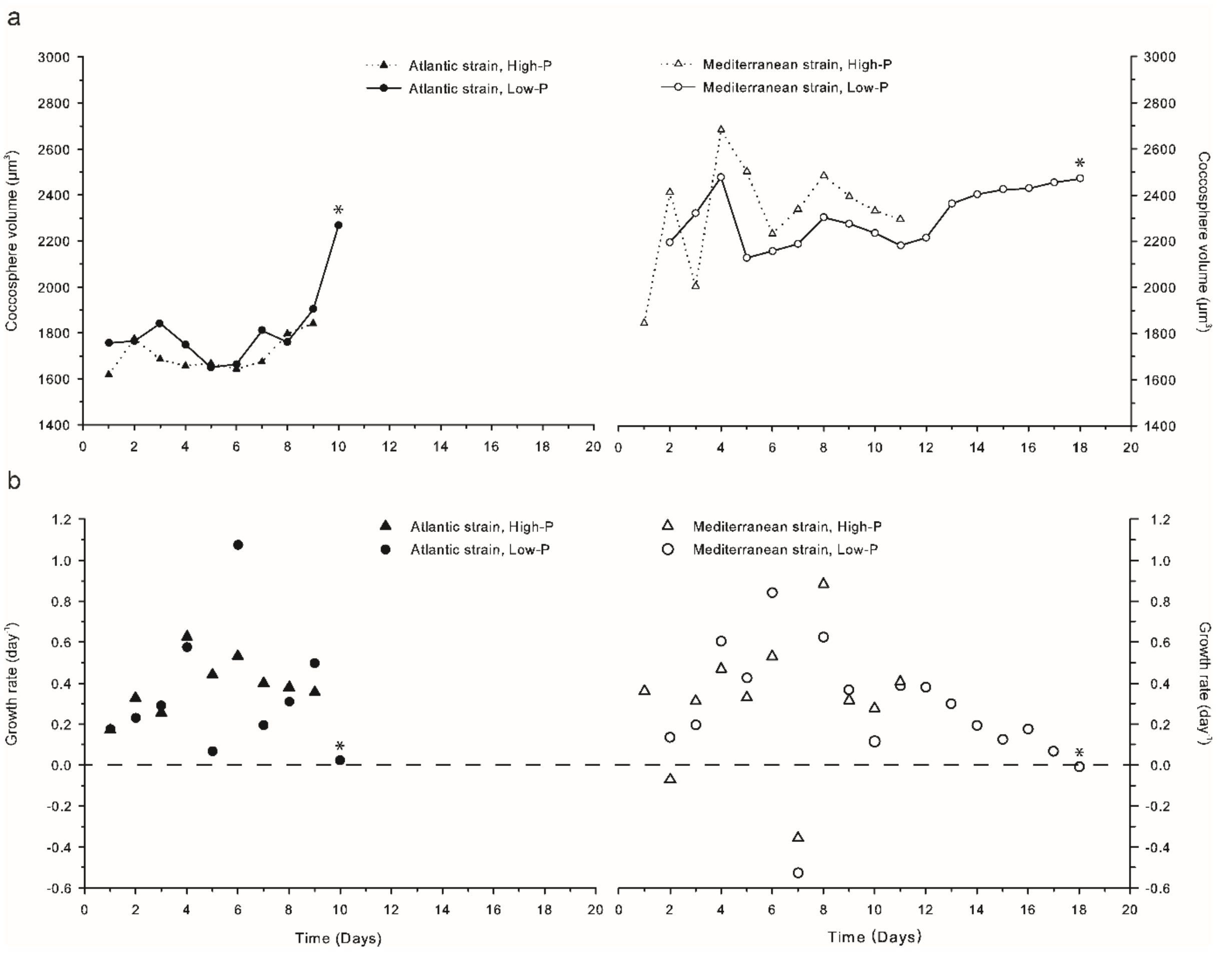

Supplementary Figure 2. Changes in coccosphere volume and incremental (day-by-day) growth rate during the course of the experiment. a: Coccosphere size measurements of both strains growing in High-P and Low-P medium (CASY measurements). Data points represent mean values of individual replicates. b: Incremental (day-by-day) growth rates of both strains growing in High-P and Low-P medium. Data points represent mean values of individual replicates. Dashed line marks the zero-growth rate. The asterisk (*) marks the sampling day of the Low-P batch cultures.

a

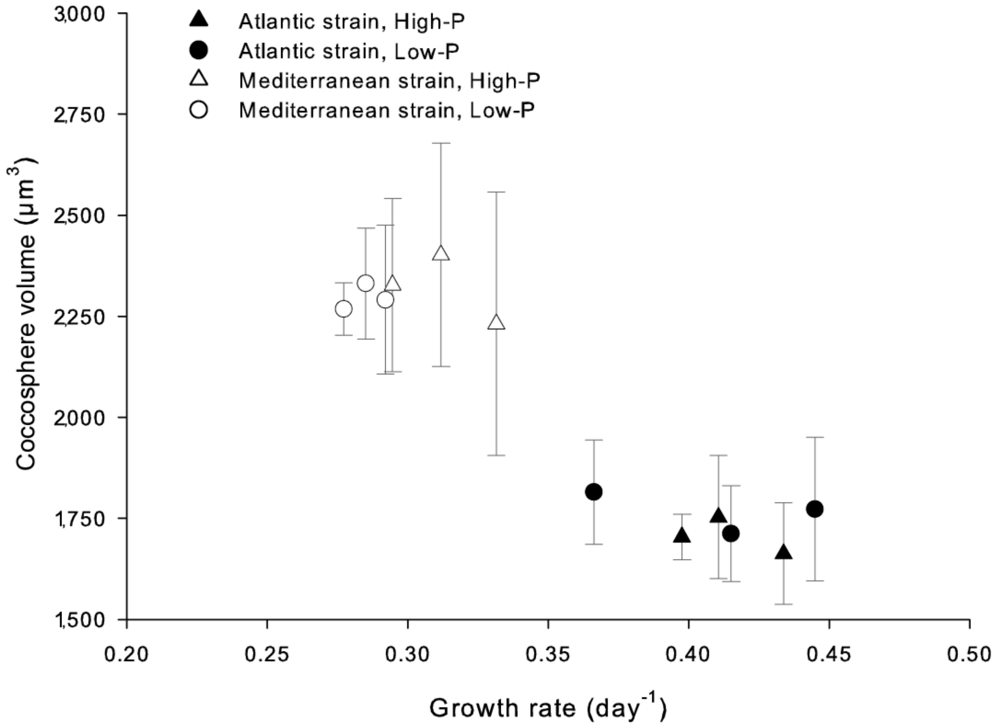

b

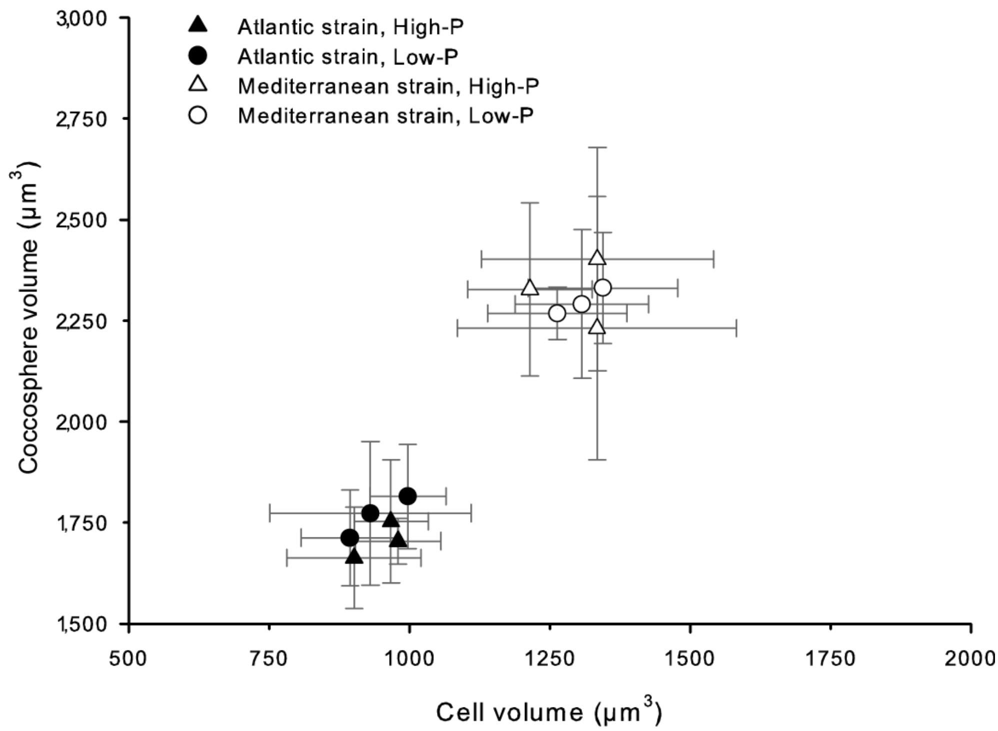

Supplementary Figure 3. Coccosphere and cell volume in relation to growth rate of *Helicosphaera carteri*. a: The relationship between coccosphere volume (CASY measurements, ±1 SD) and growth rate of each replicate. Only data collected during exponential growth is included. b: Coccosphere volume and cell volume of both strains during exponential growth. Data points represent mean values of individual replicates (N=3). Error bars show ±1 SD.

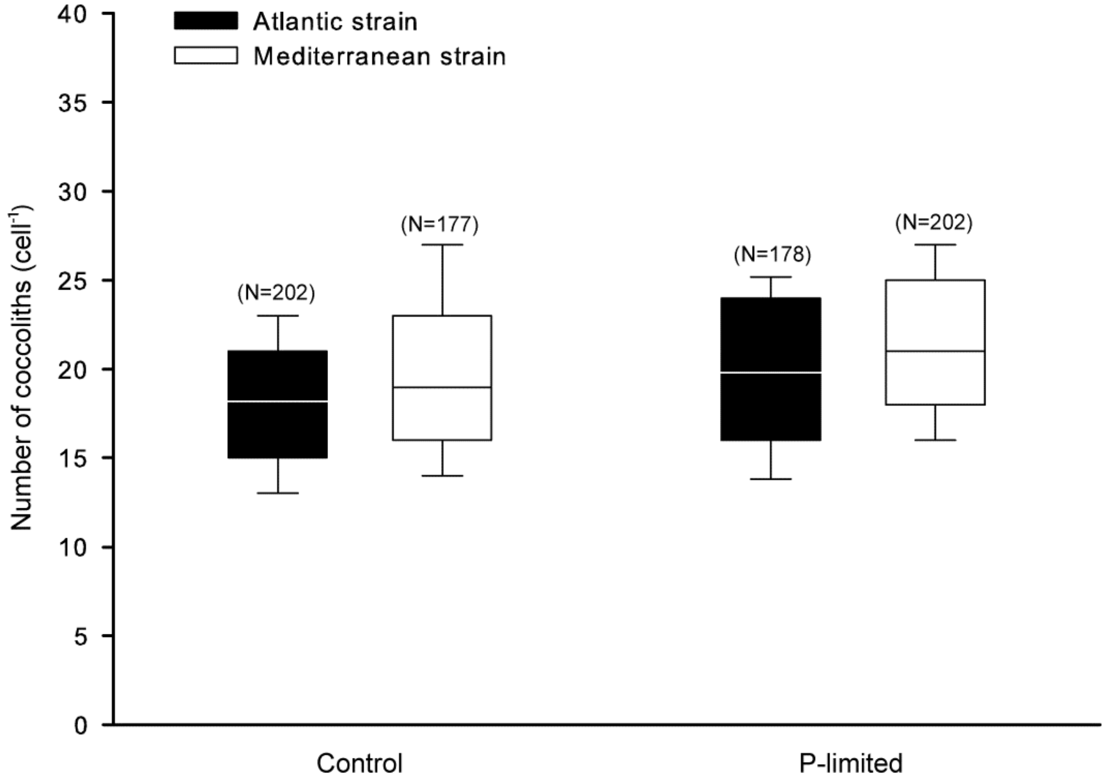

Supplementary figure 4. Number of coccoliths per cell for *Helicosphaera carteri*. Each box plot represents pooled measurements from three replicates, with the line indicating the mean value and the bars showing minimum and maximum.

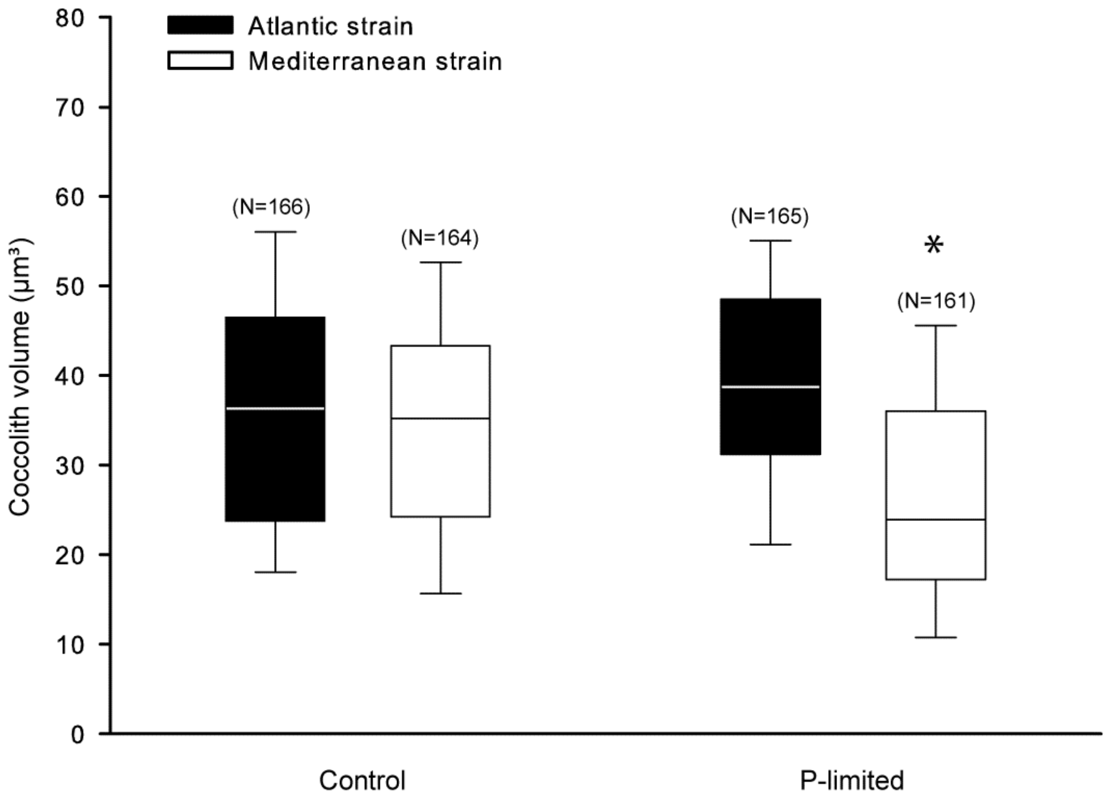

Supplementary Figure 5. Coccolith volume measurements. Each box plot represents pooled measurements from three replicates. The lines indicate mean values and the bars show minimum and maximum values. The asterisk (*) highlights the batch culture with 2.6-fold higher cell density and altered carbonate chemistry.

Supplementary Table 1. *Helicosphaera carteri* strains investigated in a preliminary investigation and two strains marked with an asterisk (*) that were compared in the final experiment. Values indicate mean coccosphere and cell volume of 3 replicate batches. Values in brackets show ±1 SD (N=3).

RCC #	Strain ID	Isolation site	Isolation date	GPS coordinates	Isolated by	Coccosphere volume (µm³)	Cell volume (µm³)	Accession number
1333	AC428	South Atlantic Ocean (Southern Benguela upwelling)	1/9/2000	S 34° 28' E 17° 18'	Ian Probert	1403 (±50)	792 (±17)	n/a
1329	AC421	South Atlantic Ocean (Southern Benguela upwelling)	1/9/2000	S 36° 38' E 16° 54'	Ian Probert	1366 (±90)	776 (±62)	n/a
1323*	AC419	South Atlantic Ocean (Southern Benguela upwelling)	1/9/2000	S 37° 40' E 14° 50'	Ian Probert	1646 (±128)	785 (±36)	KT626608
1334*	VF13	Western Mediterranean Sea (Villefranche-sur-Mer Bay)	15/9/2007	N 43° 41' E 07° 19'	Ian Probert	2080 (±80)	1248 (±159)	KT626609

Supplementary Table 2. Mean cell density, growth rate, elemental quotas and production rates measured at the end of the experiment. The asterisk (*) highlights the batch culture with 2.6-fold higher cell density and altered carbonate chemistry. Values in brackets show ±1 SD (N=3).

	**Cell density (cells ml^{-1})**	**Growth rate (day^{-1})**	**POP quota (pg $cell^{-1}$)**	**POP production (pg $cell^{-1}day^{-1}$)**	**PON quota (pg $cell^{-1}$)**	**PON production (pg $cell^{-1}day^{-1}$)**	**POC quota (pg $cell^{-1}$)**	**POC production (pg $cell^{-1}day^{-1}$)**	**PIC quota (pg $cell^{-1}$)**	**PIC production (pg $cell^{-1}day^{-1}$)**	**PIC/POC production**
**Atlantic strain**											
High-P	16,249 (±1207)	0.41 (±0.01)	11.73 (±1.24)	4.84 (±0.36)	24.04 (±4.92)	9.94 (±1.97)	123.61 (±31.78)	51.23 (±13.26)	277.63 (±67.11)	114.62 (±26.39)	2.29 (±0.41)
Low-P	15,590 (±82)	0.41 (±0.03)	8.03 (±0.72)	n/d	33.22 (±5.82)	n/d	177.5 (±51.30)	n/d	384.25 (±120.10)	n/d	2.30 (±0.86)
**Mediterranean strain**											
High-P	15,841 (±2421)	0.31 (±0.02)	13.17 (±1.31)	4.13 (±0.54)	23.25 (±6.22)	7.26 (±1.96)	160.56 (±44.35)	50.21 (±14.28)	289.79 (±73.05)	90.42 (±22.06)	1.82 (±0.18)
Low-P*	40,781 (±1856)	0.28 (±0.01)	3.26 (±0.16)	n/d	18.13 (±4.32)	n/d	235.47 (±43.56)	n/d	378.67 (±94.61)	n/d	1.59 (±0.14)

Supplementary Table 3. Medium chemistry and basic carbonate chemistry parameters measured at the end of the experiment. The asterisk (*) highlights the batch culture with 2.6-fold higher cell density and altered carbonate chemistry. Values indicate means of 3 replicate batches. Values in brackets show ±1 SD (N=3).

	**Residual phosphate (μM)**	**Total alkalinity (μmol kg^{-1})**	**pH NBS**	**DIC (μmol kg^{-1})**	**Ω_{Ca}**
**Atlantic strain**					
High-P	7.74 (±0.8)	1321 (±65)	7.87 (±0.13)	1220 (±62.34)	1.54 (±0.23)
Low-P	0.13 (±0.03)	1275 (±63)	7.96 (±0.07)	1159 (±45.90)	1.78 (±0.24)
**Mediterranean strain**					
High-P	8.19 (±0.22)	1433 (±47)	7.99 (±0.04)	1292 (±49.91)	2.12 (±0.07)
Low-P*	0.14 (±0.03)	708 (±19)	7.86 (±0.03)	639 (±7.40)	0.79 (±0.05)
